# A Case of Thrombocytopenia, Anasarca (Edema, Pleural Effusion, and Ascites), Fever, Reticulin Fibrosis/Renal Dysfunction, and Organomegaly (TAFRO) Syndrome Initially Not Presenting With Thrombocytopenia: A Role of Immature Platelet Fraction

**DOI:** 10.7759/cureus.58772

**Published:** 2024-04-22

**Authors:** Mutsuo Kanda, Koichi Kitamura, Akira Saito, Koichi Hayashi, Toshihiko Suzuki

**Affiliations:** 1 Department of Nephrology, Tokyo Medical University, Tokyo, JPN; 2 Department of Nephrology, Diabetes, and Endocrinology, Tokyo Bay Urayasu Ichikawa Medical Center, Urayasu, JPN; 3 Department of Pathology, Tokyo Bay Urayasu Ichikawa Medical Center, Urayasu, JPN; 4 Department of Emergency and Critical Care Medicine, St. Marianna University School of Medicine, Kawasaki, JPN

**Keywords:** acute kidney injury, hematology laboratory, immature platelet fraction, thrombocytopenia, tafro syndrome

## Abstract

Thrombocytopenia, anasarca (edema, pleural effusion, and ascites), fever, reticulin fibrosis/renal dysfunction, and organomegaly (TAFRO) syndrome is a rare and severe systemic disease. The emergence of thrombocytopenia, however, may be preceded by other signs or symptoms, which could delay the diagnosis of the disease. We reported a case in which an increased immature platelet fraction (IPF), a surrogate marker for megakaryocytic activity, preceded the development of thrombocytopenia, and finally, we diagnosed the patient with TAFRO syndrome.

A 79-year-old male with a previous history of uninephrectomy due to bladder and ureteral cancer was admitted to our hospital because of massive edema and progressive impairment in renal function. On admission, inguinal lymphadenopathy, elevated C-reactive protein (CRP), bilateral pleural effusion, and ascites were observed, and the lymph node biopsy showed that atrophic lymphoid follicles and germinal centers were observed along with prominent glomeruloid vascular proliferation and the expansion of the interfollicular spaces consistent with the feature of Castleman’s disease. The peripheral platelet count did not reach the level of the criteria for TAFRO syndrome (13.9×10^4^/µL), but the immature platelet fraction was increased (11.6%), and bone marrow biopsy revealed hyperplasia of megakaryocytes. During the course of the preemptive treatment with prednisolone and tocilizumab, thrombocytopenia was uncovered, and the patient was finally diagnosed as having TAFRO syndrome. Thus, the present case may offer valuable information on the role of the immature platelet fraction in the establishment of the early diagnosis of TAFRO syndrome.

## Introduction

Thrombocytopenia, anasarca (edema, pleural effusion, and ascites), fever, reticulin fibrosis/renal dysfunction, and organomegaly (TAFRO) syndrome was first described in 2010 by Takai et al. in three Japanese patients who presented with this constellation of symptoms [1]. It is considered a distinct subtype of idiopathic multicentric Castleman’s disease (iMCD), known as iMCD-TAFRO. Compared to typical iMCD, TAFRO syndrome has a more acute and aggressive clinical course, with rapid deterioration and serious disease. Prompt diagnosis and treatment are important, as TAFRO syndrome can be rapidly fatal if not managed appropriately. Although the disease entity remains to be fully clarified, growing evidence suggests that the excess production of cytokines, including interleukin 6 (IL-6) and vascular endothelial growth factor, contributes to the development of TAFRO syndrome [2,3]. Conventionally, the diagnosis of this syndrome is made based on the triad (thrombocytopenia, anasarca, and fever/C-reactive protein {CRP}) and several subsidiary symptoms [4]. There have been several reports, however, showing that the platelet count did not reach a level sufficient to meet the diagnostic criteria, whereas most of the signs and symptoms appeared in a full-fledged state early during the clinical course [5-8]. These observations may raise concerns that the failure to fulfil the diagnostic criteria for TAFRO syndrome may delay the initiation of the treatment.

Although the mechanisms for thrombocytopenia in TAFRO syndrome remain undetermined, a couple of reports show an increased immature platelet fraction (IPF) prior to the diagnosis of TAFRO syndrome [9,10]. This parameter, i.e., the ratio of immature platelets versus total platelet count, is thought to reflect megakaryocyte activity [11,12] and actually is elevated in patients with immune thrombocytopenic purpura [13], thus serving as a surrogate marker for the enhanced destruction and consumption of platelets. We have experienced a case in which anasarca, increased CRP, lymphadenopathy, renal deterioration, and an elevated IPF developed. Then, a decrease in platelets to a level of the diagnostic criteria for TAFRO syndrome ensued. Although this is an unproven hypothesis, our present case suggests that IPF is considered a predictive marker in the diagnosis of TAFRO syndrome, and an elevated IPF may facilitate the establishment of an early diagnosis of the disease.

## Case presentation

A 79-year-old male with a history of hypertension (since age 50 years) and diabetes mellitus (since age 60 years) was admitted to our medical center because of general malaise and anorexia lasting two months and renal deterioration compared to two years prior to the last visit to our clinic (serum creatinine: from 2.0 in two years ago to 2.8 mg/dL on admission). He had undergone transurethral resection of a bladder tumor (76 years old) and further received a right nephrectomy/ureterectomy due to ureteral cancer (77 years old). Also, he was taking amlodipine 5 mg per day and carvedilol 10 mg per day. On admission, his body temperature was 36.7°C, blood pressure was 125/80 mmHg, heart rate was 77 beats/minute, respiratory rate was 17 breaths/minute, and oxygen saturation was 99% on ambient room air. Physical examination revealed no apparent hepatosplenomegaly, abnormal heart sound, or crackles but shifting dullness of his abdomen and palpable lymph nodes in the right groin. A mild degree of edema was observed in both extremities.

**Figure 1:**
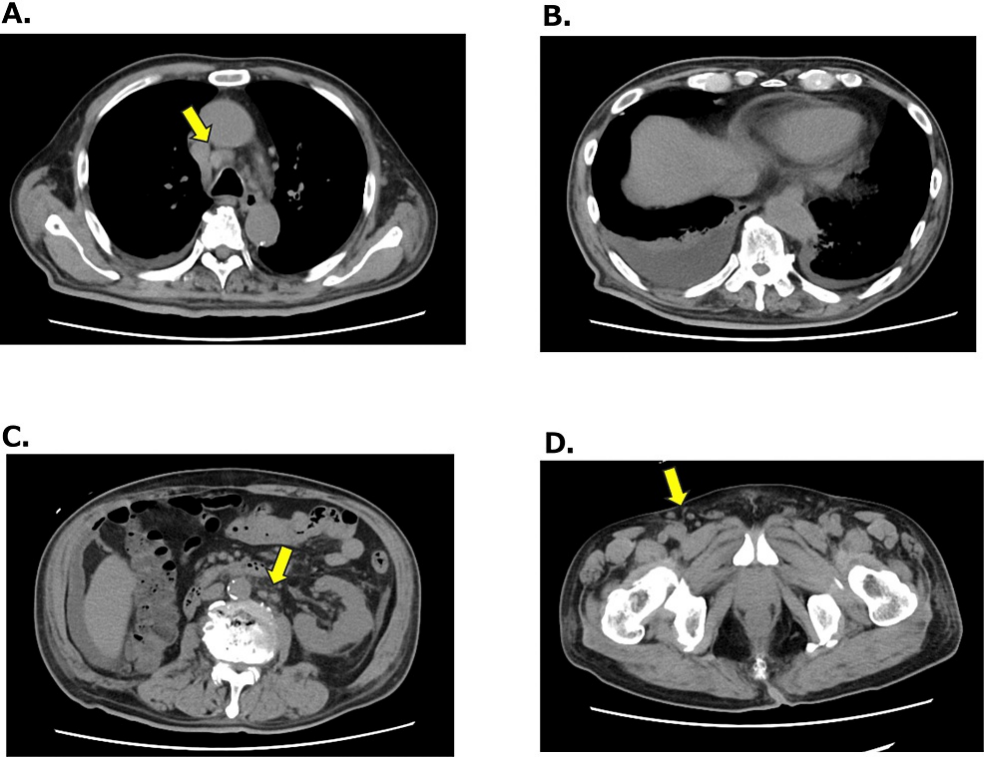
CT on admission Chest CT scan showed mediastinal lymph node swelling (A) and bilateral pleural effusion (B). The abdominal CT image demonstrated ascites and paraaortic lymph node swelling, as well as post nephrectomy (C), and lymph node swelling in the right inguinal fossa (D) CT: computed tomography

**Figure 2:**
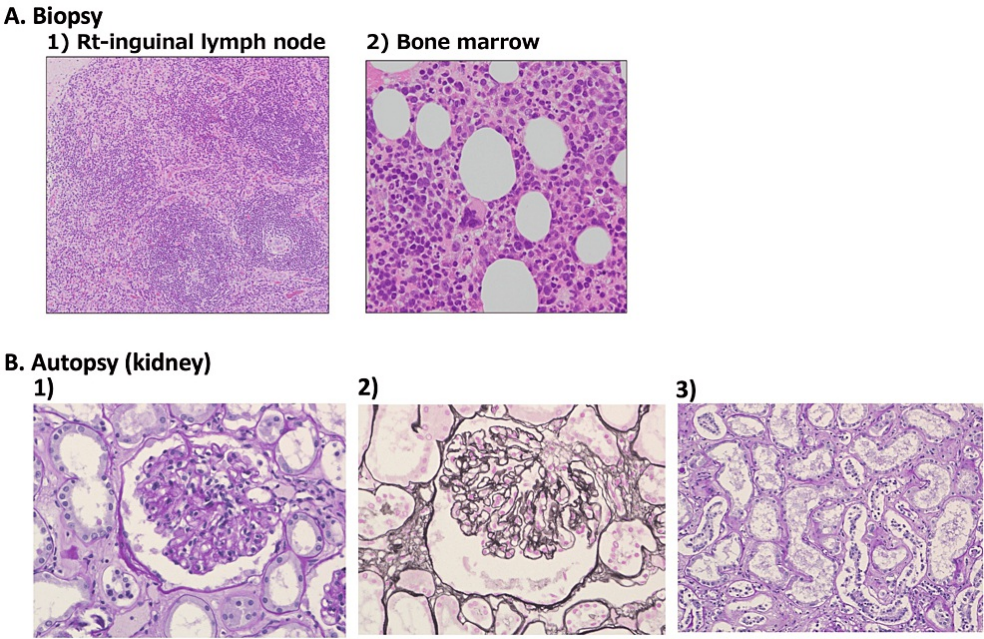
Biopsy (A) and autopsy (B) Atrophic lymphoid follicles and germinal centers were observed along with prominent glomeruloid vascular proliferation and the expansion of the interfollicular spaces (A1, hematoxylin-eosin staining, ×100). Bone marrow biopsy showed hypercellular marrow with megakaryocytic hyperplasia (A2, hematoxylin-eosin staining, ×400). Renal histopathology indicated diffuse proliferation and local aggravated endothelial cell proliferation. The basement membrane was thickened heterogeneously (B1 and B2, periodic acid-Schiff staining and periodic acid-methenamine silver staining, ×400, B3; periodic acid-Schiff staining, ×400) Rt: right

Laboratory data showed mild microcytic anemia but normal platelet counts (Table 1). Liver function tests revealed normal aspartate aminotransferase (AST)/alanine aminotransferase (ALT) but elevated alkaline phosphatase (ALP) and gamma-glutamyl transpeptidase (GGTP). Renal function was markedly impaired (estimated glomerular filtration rate {eGFR}=18 mL/minute/1.73 m^2^), and progressive proteinuria was detected (from 0.17 to 1.21 g/gram creatinine {gCr} for 28 days). Markedly elevated CRP and erythrocyte sedimentation rate (ESR) were noted without significant increases in immunoglobulins (Ig). Titers of various autoantibodies including myeloperoxidase-antineutrophil cytoplasmic antibody (MPO-ANCA), proteinase 3 (PR3)-ANCA, anti-glomerular basement membrane (GBM) antibody, anti-nuclear antibody, and anti-cyclic citrullinated peptide (CCP) antibody were normal or modestly elevated. Soluble IL-2R was markedly elevated, but the haptoglobin level did not decrease. A computed tomography scan of the chest and abdomen revealed pleural effusion and massive ascites, as well as mediastinal, paraaortic, and inguinal lymphadenopathies (Figure 1). Lymph node biopsy showed an increased number of lymphoid follicles with atrophic germinal centers compatible with the feature of Castleman’s disease of the hypervascular type (Figure 2A). Bone marrow biopsy indicated increased hematopoietic cells and megakaryocytes with no malignant appearance (Figure 2B).

**Table 1:** Laboratory data on admission SS-A, Sjögren’s syndrome-related antigen A; HPF, high-powered field; gCR, gram creatinine

Test	Result	Reference values
White blood cells (WBC)	9400/µL	40-80*×*10^2^/μL
Red blood cells (RBC)	412*×*10^4^/µL	410-530*×*10^4^/μL
Hemoglobin (Hb)	10.8 g/dL	14.0-18.0 g/dL
Mean corpuscular volume (MCV)	80.1 fL	84.0-92.0 fL
Platelets (PLT)	15.1*×*10^4^/µL	15-35*×*10^4^/μL
Total protein (TP)	6.0 g/dL	6.7-8.3 g/dL
Albumin (Alb)	2.5 g/dL	3.1-5.1 g/dL
Aspartate aminotransferase (AST)	22 U/L	13-33 U/L
Alanine aminotransferase (ALT)	7 U/L	8-42 U/L
Lactate dehydrogenase (LD)	208 U/L	119-229 U/L
Alkaline phosphatase (ALP)	205 U/L	38-113 U/L
Gamma-glutamyl transpeptidase (GGTP)	129 U/L	10-47 U/L
Total bilirubin (T-Bil)	0.88 mg/dL	0.2-1.2 mg/dL
Hemoglobin A1c (HbA1c)	6.40%	4.6%-6.2%
Blood urea nitrogen (BUN)	21.7 mg/dL	9-22 mg/dL
Creatinine (Cr)	2.76 mg/dL	0.61-1.04 mg/dL
Estimated glomerular filtration rate (eGFR)	18 mL/minute/1.73 m^2^	
Sodium (Na)	137 mEq/L	138-146 mEq/L
Potassium (K)	4.2 mEq/L	3.5-5.0 mEq/L
Chloride (Cl)	101 mEq/L	96-107 mEq/L
Calcium (Ca)	8.5 mg/dL	8.8-10.6 mg/dL
Inorganic phosphorus (iP)	3.8 mg/dL	2.4-4.5 mg/dL
C-reactive protein (CRP)	18.4 mg/dL	<0.30 mg/dL
Erythrocyte sedimentation rate (ESR)	74 mm/hour	<10 mm/hour
Complement component 3 (C3)	117 mg/dL	69-128 mg/dL
Complement component 4 (C4)	22 mg/dL	14-36 mg/dL
50% hemolytic unit of complement (CH50)	37 U/mL	30-45 U/mL
Immunoglobulin G (IgG)	1154 mg/dL	680-1620 mg/dL
Immunoglobulin G4 (IgG4)	15 mg/dL	5-117 mg/dL
Immunoglobulin A (IgA)	295 mg/dL	84-438 mg/dL
Immunoglobulin M (IgM)	55 mg/dL	57-288 mg/dL
Myeloperoxidase-antineutrophil cytoplasmic antibody (MPO-ANCA)	0.5 U/mL	<3.5 U/mL
Proteinase 3-antineutrophil cytoplasmic antibody (PR3-ANCA)	<0.6 U/mL	<2.0 U/mL
Anti-glomerular basement membrane antibody (anti-GBM Ab)	<1.5 U/mL	<7.0 U/mL
Anti-nuclear Ab (ANA)	*×*40	
Anti-SS-A Ab	<0.4 U/mL	<7.0 U/mL
Rheumatoid factor (RF)	26 IU/mL	<15.0 IU/mL
Anti-cyclic citrullinated peptides antibodies (anti-CCP Ab)	0.5 U/mL	<4.5 U/mL
Immunoelectrophoresis	Negative	(-)
Serum/urine		(-)
κ/λ ratio	0.98	0.26-1.65
Soluble interleukin-2 receptor (s IL-2R)	3482 U/mL	122-496 U/mL
Haptoglobin	225 mg/dL	19-170 mg/dL
Thyroid-stimulating hormone (TSH)	6.95 µIU/mL	0.35-4.94 µIU/mL
Free triiodothyronine (fT3)	2.82 pg/dL	1.88-3.88 pg/mL
Free thyroxine (fT4)	1.0 ng/dL	0.70-1.72 ng/dL
Adrenocorticotropic hormone (ACTH)	33 pg/mL	7.2-63.3 pg/mL
Cortisol	10.6 µg/dL	6.2-22.7 μg/dL
Hepatitis B surface antigen (HBsAg)	(-)	(-)
Hepatitis B surface antibody (HBsAb)	(-)	(-)
Hepatitis B virus antibody (HBV-Ab)	(-)	(-)
Hepatitis C virus antibody (HCV-Ab)	(+)	(-)
Hepatitis C virus-ribonucleic acid (HCV-RNA)	(-)	(-)
Human immunodeficiency virus antibody (HIVAb)	(-)	(-)
*Treponema pallidum* antibody (TP-Ab)	(-)	(-)
Rapid plasma reagin (RPR)	(-)	(-)
Interferon-gamma release assay (IGRA) T-SPOT®	(-)	(-)
COVID-19 polymerase chain reaction (COVID-19-PCR)	(-)	(-)
Blood culture	(-)	(-)
Proteinuria	(+), 0.17 g/gCr	(-)
Hematuria	(-)	(-)
Urine red blood cells (RBC)	1-4/HPF	1-4/HPF
Urine white blood cells (WBC)	1-4/HPF	1-4/HPF
N-acetyl-β-D-glucosaminidase (NAG)	33.8 U/L	<11.5 U/L

**Figure 3:**
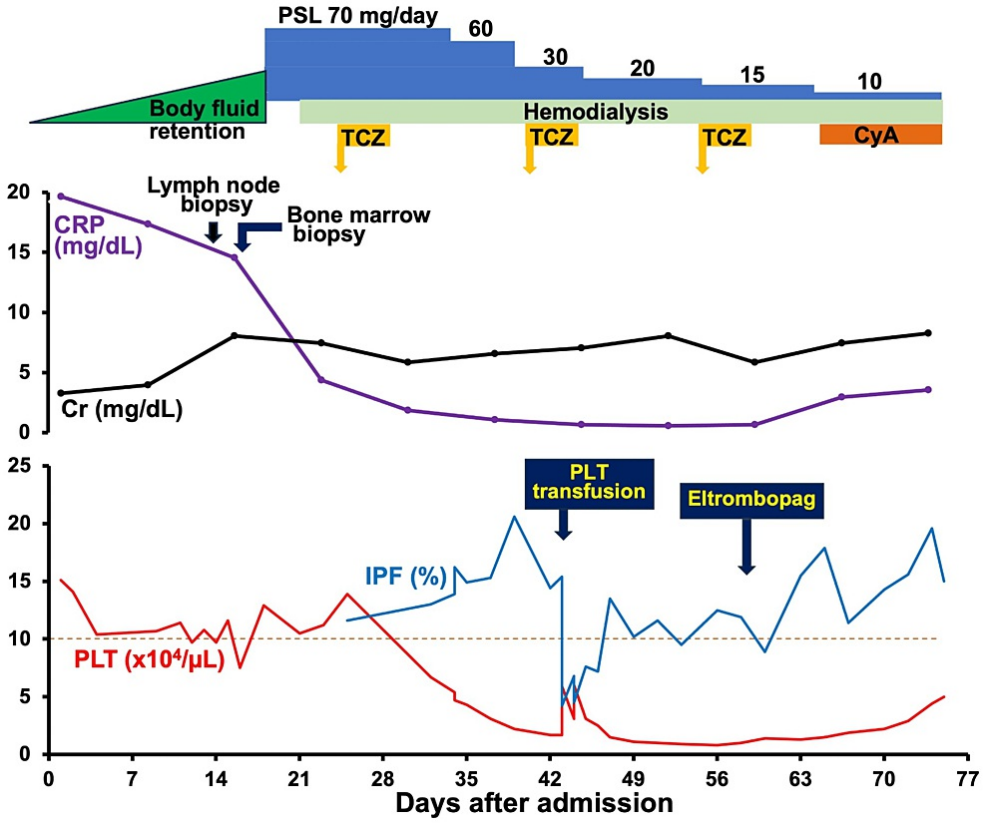
Clinical course PLT, platelet; IPF, immature platelet fraction; Cr, creatinine; PSL, prednisolone; TCZ, tocilizumab; CyA, cyclosporine A; CRP, C-reactive protein

While the platelet count did not reach the cutoff value to define TAFRO syndrome (i.e., <105/µL) in the early stages of hospitalization, an increase in the IPF was noted (Figure 3 and Table 2). Since other symptoms and laboratory data were compatible with their respective criteria for TAFRO syndrome, we initiated preemptive treatment with prednisolone 1 mg/kg/day, followed by tocilizumab 8 mg/kg, alongside intermittent hemodialysis therapy with eltrombopag 12.5 mg, but failed to obtain a favorable effect. The addition of cyclosporine A 3 mg/kg tended to increase the platelet count, but the patient died of bacterial pneumonia. At autopsy, renal histology revealed that glomerular capillary endothelial cells were markedly enlarged with a feature of double contours in capillary walls (Figure 2B).

**Table 2:** Reported cases with normal platelet count but elevated immature platelet fraction before the diagnosis of TAFRO syndrome A standard range for IPF in healthy subjects is 2.07% (mean±2 SD: 0.17-3.97) * and ^$^Estimated from the graphic data in Figure 1 [9] and Figure 2 [10], respectively ^#^Obtained in two consecutive days ^##^Obtained after successful treatment **Obtained after eltrombopag IPF, immature platelet fraction; NA, not available; TAFRO, thrombocytopenia, anasarca (edema, pleural effusion, and ascites), fever, reticulin fibrosis/renal dysfunction, and organomegaly

		Before diagnosis	At diagnosis	Follow-up
Nagai et al. [9]	Platelet (×104/µL)	17.9	8.8*	20.0*
	IPF (%)	20.7	NA	NA
Saga et al. [10]	Platelet (×104/µL)	13.7	4.4^$^	17.4$
	IPF (%)	18.2	30.0^$^	3.5^$,##^
Present case	Platelet (×104/µL)	11.2/13.9^#^	4.7	1.0
	IPF (%)	12.1/11.6^#^	16.2	11.9**

## Discussion

TAFRO syndrome is a rare disease composed of multiple nonspecific signs and symptoms, including thrombocytopenia, elevated CRP/fever, anasarca, and other symptoms such as renal impairment and lymphadenopathy. In the present case, we confirmed these symptoms and further found Castleman’s disease-like appearance in lymph node histopathology and megakaryocytic hyperplasia in the bone marrow biopsy. Other diseases such as systemic lupus erythematosus (SLE), ANCA-related disorders, malignant lymphoma, and IgG4-related disease were excluded based on the clinical symptoms and laboratory data. Based on enlarged lymph nodes and Castleman-like histopathology (hypervascular type), we therefore established the patient’s disease as iMCD-TAFRO syndrome.

Thrombocytopenia (<105/µL) constitutes an essential component in the diagnosis of TAFRO syndrome. In the present case, we observed an increased IPF before the development of thrombocytopenia and the establishment of the diagnosis (Figure 3). Recent studies have demonstrated that the IPF reflects the thrombopoietic activity and varies depending on the underlying disorders [14]. As an example, IPF is known to be elevated in patients with thrombocytopenia due to peripheral platelet destruction/consumption, such as immune thrombocytopenic purpura, and immunological mechanisms are thought to be involved [15]. Likewise, platelet’s most frequent volume, a surrogate marker for mean platelet volume and a possible parameter for platelet turnover [16,17], was also increased in this case (i.e., 11.0 fL, standard range {mean±2 SD}: 7.84-10.2 fL). Although the precise mechanism for the thrombocytopenia in TAFRO syndrome remains undetermined, both our case and a couple of previous case reports indicated that an increase in IPF preceded the development of thrombocytopenia and persisted during the active phase of the disease (Table 2 and Figure 3) [9,10]. Furthermore, Saga et al. [10] showed that a decrease in IPF was followed by the recovery from thrombocytopenia (Table 2). The changes in IPF may therefore predict the subsequent alterations in platelet counts.

The patient was initially treated with corticosteroids and hemodialysis therapy because of massive fluid retention and the acute exacerbation of renal function. Some cases of TAFRO syndrome, e.g., iMCD-TAFRO syndrome, can be treated by blocking IL-6 signaling, which has been reported to suppress inflammation and improve symptoms [18]. After the establishment of the diagnosis of TAFRO syndrome, tocilizumab therapy was added but failed to suppress the activity. It has been reported that cyclosporine A coadministered with corticosteroids effectively increases platelet counts [8,19]. In the present case, we observed that adding cyclosporine A on corticosteroid therapy tended to elevate the platelet counts though the patient died of pneumonia. Of note, eltrombopag, a thrombopoietin receptor agonist, failed to increase the platelet counts, in which the finding is consistent with a report by Sato et al. [19] and may provide a clue for elucidating the mechanism of thrombocytopenia in TAFRO syndrome. In conclusion, IPF constitutes a predictive marker in the diagnosis of TAFRO syndrome. The significance of the findings presented in the case should be confirmed in future studies.

## Conclusions

We report a case with TAFRO syndrome in which an increased IPF precedes the development of thrombocytopenia, an essential feature characterizing this syndrome. Because the IPF is routinely assessed with an automated analyzer, the diagnosis of TAFRO syndrome can be made earlier before overt thrombocytopenia develops, which may facilitate the initiation of the therapy.
